# Prevalence of middle mesial canal and radix entomolaris of mandibular first permanent molars in a western Chinese population: an in vivo cone-beam computed tomographic study

**DOI:** 10.1186/s12903-020-01218-z

**Published:** 2020-08-17

**Authors:** Xin Qiao, Hualing Zhu, Yujia Yan, Jinglin Li, Jiayin Ren, Yuan Gao, Ling Zou

**Affiliations:** 1 State Key Laboratory of Oral Diseases & National Clinical Research Center for Oral Diseases & West China Hospital of Stomatology, Sichuan University, Chengdu, China; 2 State Key Laboratory of Oral Diseases & National Clinical Research Center for Oral Diseases & Department of Radiology, West China Hospital of Stomatology, Sichuan University, Chengdu, China; 3 State Key Laboratory of Oral Diseases & National Clinical Research Center for Oral Diseases & Department of Endodontics, West China Hospital of Stomatology, Sichuan University, Chengdu, China

**Keywords:** The first permanent mandibular molar, Root canal system, Cone-beam computed tomographic (CBCT), Middle mesial canals (MMC), Radix entomolaris (RE)

## Abstract

**Background:**

The aim of this study was to investigate the prevalence of the middle mesial canal (MMC) and radix entomolaris (RE) in mandibular first permanent molars in a western Chinese population using cone-beam computed tomography (CBCT).

**Methods:**

A total of 1174 CBCT images of the mandibular first molars were collected from West China Hospital of Stomatology, Sichuan University. The following information was recorded and evaluated: the detection rate and location of the MMC and RE, the curvature of the RE, the canal configuration and bilateral symmetry.

**Results:**

The detection rates of the MMC and RE were 3.41 and 25.04%, respectively, as calculated by individuals, and 1.79 and 22.15%, respectively, as calculated by total teeth. The average curvature in the buccolingual (BL) orientation (40.63 ± 14.39°) was significantly larger than that in the mesiodistal (MD) orientation (17.64 ± 7.82°) (*p* < 0.05). Of 587 patients, 71.72% (421/587) had bilateral symmetry according to the root canal morphology. The prevalence of three-rooted mandibular first molars was higher in males than in females, while the prevalence of two-rooted mandibular first molars was higher in females than in males.

**Conclusions:**

Our results showed that the RE could be detected in almost 1/4 of the western Chinese population; thus, RE detection requires special attention and careful assessment in endodontic treatment.

## Background

It is well known that successful endodontic treatment depends on many factors, among which the anatomy of the teeth and root canal system provides the anatomical basis for endodontic treatment [1, 2]. Missing a single root canal in endodontic treatment may lead to secondary or persistent apical periodontitis [3]. Mandibular first molars, the first permanent teeth that appear in our oral cavities, which are defined as “the key of the occlusion” and have a large number of pits and fissures on the occlusal surface, demonstrate a poor self-cleaning effect. Therefore, the mandibular first molars with a high risk of caries are the most likely to undergo endodontic treatment [4–6]. Traditionally, the most common form of a mandibular first molar has been briefly depicted as a two-rooted tooth with two canals in the mesial root and one or two canals in the distal root [1]. However, there are several aberrant canal morphology in the mandibular molars, and the most common variations include an additional distolingual root and canal [7] and a third canal in the mesial root termed as the middle mesial canal (MMC) [8]. Since the missing canals ay contain necrotic debris, tissue remnants, or organic substrates that facilitate the growth of microorganisms, it is necessary to locate all root canals, especially MMC and distolingual root canals, debride them thoroughly and prevent reinfection, to avoid failure of endodontic treatment.

Vertucci and William [9] as well as Barker et al. [10] first reported the presence of the MMC in mandibular molars in 1974. Since then, there have been multiple studies that have reported this kind of variation in the mesial root between the mesiobuccal canal (MBC) and mesiolingual canal (MLC), named the “intermediate”, “mesiocentral”, “middle mesial”, “third mesial” or “accessory mesial” root canal [1, 11–15]. Previous studies have reported a frequency of this canal ranging from 0.26–46.15%, and this extra canal has received special attention when endodontic treatment is needed [16, 17].

The presence of mandibular first molars with an third distolingual root was noticed earlier than the presence of the MMC, which was first reported in 1844 by Carabelli et al. [18]. It has been found that the additional distolingual root termed the “radix entomolaris” (RE) is basically smaller and more curved than the mesiobuccal and distobuccal roots [19, 20]. Relevant literature [7, 21–25] demonstrates that the RE detection rate has an obvious genetic and ethnic predilection: A maximum of 3% is discovered in African populations, whereas the frequency is less than 5% in Eurasian, Indian, European or Caucasian populations. In populations with Mongolian descent such as the Chinese, Malaysian, Eskimo, and American Indian populations, the frequency is higher than 5% (even up to 40%). In the populations mentioned above, the RE is considered to be a kind of normal racial structure rather than a variation. We all know that western China is a multiple ethnic region, therefore, it is of great clinical value to study the RE detection rate in this region representative of China.

The purpose of the study was to analyze the root number and morphology, the canal number and configuration, and the location of the MMC and RE in the first mandibular molars in a western Chinese population in order to provide additional imaging anatomical data analysis for clinicians and improve the success rate of endodontic treatment. The symmetry of homonym teeth and whether sex was related to root canal variation were also recorded.

## Methods

### Patients

Sample calculation was based on single sample rate calculation formula: n = ($\frac{Z_{\alpha }}{\delta }$)^2^π(1-π) [26]. The overall middle mesial canal prevalence π = 69.6%, α = 0.05, δ = 0.05, one-tailed, where π is from previous studies [27] using 95% confidence intervals. To get a higher precision, we have enlarged the result calculated by the above formula by 10% as the final minimized sample size, which is 357. As a retrospective cross-sectional study, the cone-beam computed tomographic (CBCT) data were enrolled at the department of radiology of West China Hospital of Stomatology, Sichuan University from October 2017 to March 2018, from patients who have medical treatment needs (pulpitis, periapical periodontitis, dental trauma, cracked tooth, vertical root fracture, etc.) and first visit the department of endodontics. The present study initially included 1549 patients, excluding 962 patients, and ended up with 587 patients (237 males and 350 females) and 1174 bilateral mandibular first permanent molars, and the basic information of the patient’s name, gender and age was recorded. The study was approved by the Medical Ethics Committee of West China Stomatological Hospital of Sichuan University with the approval number: WCHSIRB-ST-2017-85.

The study screened teeth according to the following criteria, for the following factors might affect the accurate judgment of the root and canal configuration:

1. No trauma or defects;
2. No periapical lesions;
3. No root canal treatment or post- or crown restoration;
4. No root canals with open apices, resorption or calcification;
5. Good-quality CBCT images.

### Radiographic techniques

CBCT images were obtained using a CBCT device (3-dimension Accuitomo, J.MORITA MFG.CORP.Kyoto Japan), with those exposure parameters: 85 kvp, 4.5 mA, 17.5 s scan time, with voxel size of 0.125 mm and field of view of 60*60 mm for all images. All images were produced by an experienced technician according to the manufacturer’s instructions using lowest dose radiation.

### Evaluation of the images

The i-Dixel software (One Volume Viewer 1.5.0) was used to reconstruct and measure the image. Two endodontists concurrently analyzed all the images to reach an agreement on the findings of these images. An oral radiologist provided guidance when necessary. The root canal system of the first permanent mandibular molars was observed from the medullary cavity to the root apical layers on the coronal, sagittal and cross-section views, and the following data of the left and right sides were measured and recorded: the root number and morphology, the canal number and configuration, the prevalence of the MMC and the RE root canal, and the curvature of the RE in the buccolingual (BL) direction and in the mesiodistal (MD) direction. The methods of Schneider [28] was used to measure the angle of the RE curvature.

SPSS 21.0 software (SPSS, Inc., Chicago, IL, USA) was used for statistical analysis. Descriptive statistics was used to describe the number of different roots and root canals, as well as the detection rate of root canal morphology. The number of roots and canals, root canal morphology and detection rate of the MMC and RE in different groups according to sex and their bilateral symmetries were analyzed using the Chi-square test. Further, the curvature of the RE root in the buccolingual and mesiodistal directions were compared. Statistically significant differences were defined at *p* < 0.05.

## Results

### The detection rates and locations of the MMC and RE

**Fig. 1:**
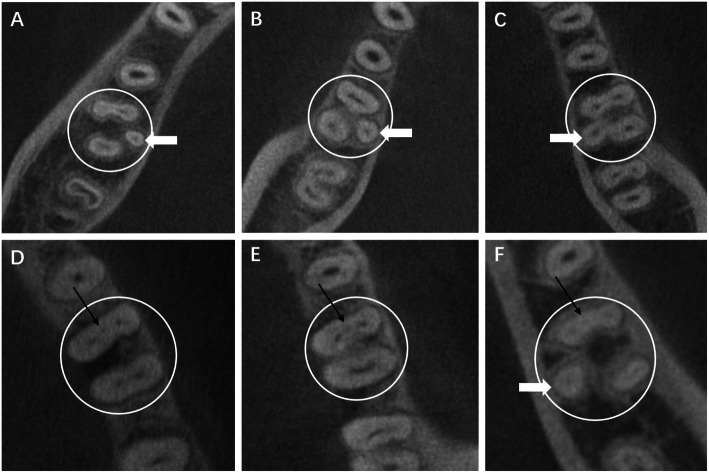
Cases of mandibular first molars with RE or MMC in the axial section; the white circles indicate the examined tooth; the white arrows indicate REs; the black arrows indicate MMCs. CBCT images show (A-C) mandibular first molars with RE, (D-E) mandibular first molars with MMC, (F) mandibular first molars with both RE and MMC

Cases of mandibular first molars with RE or MMC in the axial section are showed in Fig. 1. The detection rates of the MMC and RE were 3.41 and 25.04%, respectively, as calculated by individuals, and 1.79 and 22.15%, respectively, as calculated by the total teeth. The mean distances between the MMC and MBC orifice and between the MMC and MLC orifice were 1.84 mm and 1.95 mm, respectively, while the mean distances between the RE canal orifice and distobuccal canal orifice and between the RE canal orifice and mesiolingual canal orifice were 4.22 mm and 4.01 mm, respectively.

### The curvature of the RE

The curvature of the RE canal was more severe in the BL orientation (40.63 ± 14.39°) than in the MD orientation (17.64 ± 7.82°) (*p* < 0.05). Most RE canals exhibited severe curvature in the BL orientation and moderate curvature or a straight appearance in the MD orientation.

### The bilateral symmetry

The numbers of roots and root canals are listed in Table 1, and the canal configurations according to Vertucci’s classification are listed in Table 2.

**Table 1:** The number of roots and root canals in the mandibular first molars

Root number	Two root canals	Three root canals	Four root canals	Five root canals	Total
Two roots	53	619	219	3	894 (76.15%)
Three roots	0	44	231	5	280 (23.85%)
Total	53 (4.51%)	663 (56.47%)	450 (38.33%)	8 (0.68%)	1174 (100%)

**Table 2:** The canal configuration based on Vertucci’s classification in the mandibular first molars

Root	Type I (1)	Type II (2–1)	Type III (1–2-1)	Type IV (2–2)	Type V (1–2)	Type VI (2–1-2)	Type VII (1–2–1-2)	Type VIII (3)
Mesial root	63 (5.37%)	235 (20.02%)	48 (4.09%)	773 (65.84%)	26 (2.21%)	8 (0.68%)	0 (0)	21 (1.79%)
Distal root	918 (78.19%)	41 (3.49%)	12 (1.02%)	180 (15.33%)	20 (1.70%)	3 (0.26%)	0 (0)	0 (0)

Of the 587 patients, 91.14% (535/587) had bilateral symmetry in terms of the number of roots, 84.50% (496/587) had bilateral symmetry in terms of the number of root canals, and 71.72% (421/587) had bilateral symmetry in terms of the root canal morphology. The right mandibular first molar had a higher prevalence of having two roots (80.4%), while the left molar had a higher prevalence of having three roots (28.1%). The prevalence of the mandibular first molar with five canals was higher on the left side (3.2%) than on the right side (1.2%).

In addition, the frequency of bilateral occurrence of the MMC was only 0.05%, with no significant differences between the right and left sides. However, the frequency of bilateral occurrence of the RE was 76.87%, and there was a statistically significant difference between the right and left sides (*p* < 0.05), as it was higher on the left side.

### The association of these parameters with sex

The frequency distribution and percentage of the number of roots and root canals according to sex are listed in Table 3.

**Table 3:** The frequency distribution and percentage of the number of roots and root canals according to sex in the mandibular first molars

	Number of roots			Number of root canals				
	Two roots	Three roots		Two root canals	Three root canals	Four root canals	Five root canals	
Male (n/%)	345 (72.8%)	129 (27.2%)	474 (100.0%)	6 (1.3%)	256 (54.0%)	210 (44.3%)	2 (0.4%)	474 (100.0%)
Female (n/%)	549 (78.4%)	151 (21.6%)	700 (100.0%)	47 (6.7%)	407 (58.1%)	240 (34.3%)	6 (0.9%)	700 (100.0%)
	894 (76.1%)*	280 (23.9%)*	1174 (100.0%)	53 (4.5%)*	663 (56.5%)	450 (38.3%)*	8 (0.7%)	1174 (100.0%)

*p < 0.05, chi-square test

The prevalence of three-rooted mandibular first molars was higher in males (27.2%) than in females (21.6%), while the prevalence of two-rooted mandibular first molars was higher in females (78.4%) than in males (72.8%). The prevalence of four-canaled molars was higher in males (44.3%) than in females (34.3%), while the prevalence of two-canaled molars was higher in females (6.7%) than in males (1.3%). Except two-canaled and four-canaled molars mentioned above, the prevalence of the remaining numbers of root canals, such as three-canaled and five-canaled molars, had no significant difference in different sexes. In addition, the prevalence of the RE (Chi-square *P* = 0.273) and MMC (Chi-square *P* = 0.668) was not significantly different between males and females.

## Discussion

The most common form of a mandibular first molar is the presence of two roots with three canals: two canals located in the mesial root and one canal in the distal root. There is little difference between countries and ethnic groups [5, 29–33]. In our study, the most common root canal configuration of the mandibular first molar was type IV (65.84%) in the mesial roots and type I (78.19%) in the distal roots. The result is in accordance with the findings of most earlier studies [5, 16, 29, 31–37].

As mentioned before, the detection rates of the MMC in mandibular first molars varied among studies, ranging from 0.26–46.15% [16, 17]. This varied detection rates can be due to the varying ethnic groups and ages as well as the study design or methods of detection. In China, this anatomical variation has been found to range from 0.8 to 22% [38–41]. In the present study, the detection rate of the MMC was only 3.41%. The mean distances between the MMC and MBC orifice and the MMC and MLC orifice were 1.84 mm and 1.95 mm, respectively. These distances were obviously longer than those detected by Versiani et al. [13], who found that the average distances were 1.35 mm and 1.34 mm, respectively. In addition, Akbarzadeh et al. [42] showed that in molars with MMC, the mean distance between the MBC and MLC orifice was 3.1 mm, while the distance was 3.7 mm in those without MMC; But in a most recent study, [43] that the mean distance between the mesial canals was 3.643 mm in teeth with MMC, and 3.818 mm in teeth without MMC, and Weinberg et al. suggested that there is no significant difference between above-mentioned distance in molars with or without MMC. But these distances are also apparently shorter than those detected in our study. Therefore, clinicians can carefully explore the MMC within a wide range between the MBC and MLC to avoid missing root canals in western Chinese population as well as in Chinese populations. In addition, the analysis of data by Nosrat et al. [8] showed that the prevalence of MMC among different age groups has significant difference. They found that the prevalence of MMC was 32.1, 23.8, 3.8% in patients ≤20 years old, 21–40 years old, and > 40 years old, respectively. This finding suggests that clinicians should be more careful and spend more time searching for an MMC when handling younger patients.

In the clinic, MMCs are difficult to find and treat. When working without magnification, it is likely that the MMCs will be missed because their access always be hidden by the secondary dentin. With the use of some adjunctive aids, such as operating microscope, ultrasonic troughing and CBCT, dental clinicians were greatly facilitated in the location and treatment of MMC [40, 44]. The operating microscope and ultrasonic tip can be used for removal of any protuberance from the mesial axial wall, which would prevent direct access to the developmental groove between MBC and MLC orifice. In addition to the various diagnostic aids, operator experience has also been identified as a key factor in locating these aberrant canals. The clinician should be aware of the incidence of this type of variation in the mandibular first molar tooth and perform a preoperative radiological assessment from different angles, a proper access preparation, and thorough examination of the pulp chamber to locate and debride all the canals [45].

Versiani et al. [13] reported that the mean minor diameter of the MMC (0.16 mm) orifice was significantly smaller than that of the MBC (0.46 mm) and MLC (0.50 mm) orifices; it was always too small to detect and was also prone to root strip perforation during instrumentation. In addition, according to Akbarzadeh et al. [42], there could be an isthmus in 87% of mandibular first molars with an recognizable MMC. Due to the existence of an isthmus in most cases with an MMC, it is difficult to completely clean up the microbial biofilm attached to the isthmus, which can lead to failure in endodontic treatment [27]. Interestingly, Tahmasbi et al. [27] also found that an MMC originated from a separate apical foramen in only 2.4% of the total cases. They proposed that the omission of an MMC in mandibular molars may not be severe and would not definitely contribute to the failure of endodontic treatment in contrast to the omission of a second MB canal in maxillary molars [46].

In terms of the RE, a previous study by Zhang et al. [47] in western Chinese population, in which CBCT images of 232 mandibular first molars were detected, showed a detection rate of 30% for distal extra roots, and all of the extra roots had a type I configuration. Another study in western Chinese population [34] found that of the 558 CBCT images of mandibular first permanent molars, 24.7% exhibited a distolingual root. The results of the present study showed a detection rate of 22.15% (260/1174) for the total teeth and a detection rate of 25.04% (147/587) for individuals, which is consistent with the results described above. These results suggest that the prevalence of a RE in western Chinese populations is relatively high, and one out of every four people may have a distolingual root. Therefore, clinicians should carefully and consciously explore the presence of distolingual roots during endodontic treatment.

According to Schneider’s method, Chen et al. [25] found that most REs had a more severe curvature in the BL orientation (36.35 ± 9.38°) than in the MD orientation (9.24 ± 6.10°). The present study evaluated 487 mandibular first molars with an RE using Schneider’s method and showed that the angles of the RE root curvature in the BL and MD orientations were 40.69 ± 14.37° and 17.58 ± 7.84°, respectively; it was also found that most RE canals exhibited severe curvature in the BL orientation and exhibited moderate curvature or a straight appearance in the MD orientation, which is in agreement with previous studies. It is usually known that there is an increased risk of instrument fracture with an increasing angle of curvature [48–50]. Attention should be taken to avoid instrument fracture or perforation for the treatment of this additional unexpected canal since they are usually short and severely curved. The present study also found that the mean inter-orifice distances from the DLC to the DBC and from the DLC to the MLC were 4.22 and 4.01 mm, respectively. These distances are greater than those reported by Zhang et al. [31], in which the distances found between the two distal canal orifices in most (65.2%) mandibular first molars with an RE were 2.5–3.5 mm. This suggests that clinicians could try to explore the presence of an orifice of the RE canal within a larger range in western Chinese populations, and a highly trapezoidal cavity may be helpful for locating the RE canal orifice [51].

It is also necessary to learn the similarity and symmetry of the number and morphology of roots and root canals between the left and right sides if bilateral mandibular first molars are to be treated. Of the 587 patients we studied, 91.14% had bilateral symmetry in terms of the number of roots, 84.50% had bilateral symmetry in terms of the number of root canals, and 71.72% had bilateral symmetry in terms of the root canal morphology (both sides had the same canal configuration according to Vertucci’s classification in each root). In addition, our study showed that the right molars had a higher prevalence of having two roots (80.4%), while the left molars had a higher prevalence of having three roots (28.1%), which is consistent with the results of Wang et al. [34]. In addition, the frequency of bilateral occurrence of the MMC was only 0.05%, with no significant differences between the right and left sides. The frequency of bilateral occurrence of the RE was 76.87%, and there was a statistically significant difference between the right and left sides. The prevalence of RE according to the side of occurrence still has some dispute. Some studies have found a right-side predominance [24, 52, 53], whereas other investigators have reported REs seen more frequently on the left side [34, 54–56]. The results of the current study support the latter evidence. These contradictory findings might be attributed to the ethnic backgrounds, sample sizes and methods used.

In our study, Vertucci’s Classification has some limitations. If there was any other type of canal configuration which doesn’t fits in these eight variations, it will be embarrassing to categorize. For this reason, a more comprehensive classification such as Ahmed et al.’ s classification [57] could be introduced into further research. CBCT provides a reliable support for the clinician to have a more thorough understanding of the anatomic structures of root canal systems, especially those tiny, undetectable structures, like MMC and RE canals. As the results of our study shown, only 20 of the 587 patients detected MMC, and it doesn’t make much sense to subdivide the age as a confounder for statistical analysis. Therefore, more patients with MMC need to be included for further study about the association between MMC and age. The prevalence of RE in western Chinese populations is relatively high, and one out of every four people may have a distolingual root. And the frequency of bilateral occurrence of the RE was 76.87%. Considering the prevalence and symmetry of the RE, clinicians should pay more attention to diagnose and treat this variation.

## Conclusions

In western Chinese populations, there are 3 or 4 root canals in most mandibular first permanent molars. Vertucci type I canal configurations was most prevalent in the distal while type IV was the most prevalent in the mesial roots. The detection rates of the MMC and RE were 3.41 and 25.04%, respectively. Therefore, clinicians should be careful enough to avoid overlooking the possible existence of a RE or an MMC.

## Data Availability

All the datasets used and analyzed during the current study are available from the corresponding author on reasonable request.

## Abbreviations

MMC: Middle mesial canal
RE: Radix entomolaris
CBCT: Cone-beam computed tomography
BL: Buccolingual
MD: Mesiodistal
MBC: Mesiobuccal canal
MLC: Mesiolingual canal
